# *FGFR1* is a potential therapeutic target in neuroblastoma

**DOI:** 10.1186/s12935-022-02587-x

**Published:** 2022-04-29

**Authors:** Flora Cimmino, Annalaura Montella, Matilde Tirelli, Marianna Avitabile, Vito Alessandro Lasorsa, Feliciano Visconte, Sueva Cantalupo, Teresa Maiorino, Biagio De Angelis, Martina Morini, Aurora Castellano, Franco Locatelli, Mario Capasso, Achille Iolascon

**Affiliations:** 1 CEINGE Biotecnologie Avanzate, Via Gaetano Salvatore, 486, 80145 Naples, Italy; 2 Dipartimento di Medicina Molecolare e Biotecnologie Mediche, Università degli Studi di Napoli Federico II, 80145 Naples, Italy; 3 European School of Molecular Medicine, Università Degli Studi di Milano, 20122 Milan, Italy; 4 Hematology/Oncology and Cell and Gene Therapy Department, IRCCS Bambino Gesù Children’s Hospital, 00165 Rome, Italy; 5 Laboratory of Molecular Biology, IRCCS Istituto Giannina Gaslini, 16147 Genoa, Italy; 6 Paediatric Haematology/Oncology Department, IRCCS Bambino Gesù Children’s Hospital, 00165 Rome, Italy; 7 IRCCS Bambino Gesù Children’s Hospital, Sapienza, University of Rome, 00165 Rome, Italy

**Keywords:** *FGFR1*, Metastasis, Mutation, Neuroblastoma, Targeted therapy

## Abstract

**Background:**

*FGFR1* regulates cell–cell adhesion and extracellular matrix architecture and acts as oncogene in several cancers. Potential cancer driver mutations of *FGFR1* occur in neuroblastoma (NB), a neural crest-derived pediatric tumor arising in sympathetic nervous system, but so far they have not been studied experimentally. We investigated the driver-oncogene role of *FGFR1* and the implication of N546K mutation in therapy-resistance in NB cells.

**Methods:**

Public datasets were used to predict the correlation of *FGFR1* expression with NB clinical outcomes. Whole genome sequencing data of 19 paired diagnostic and relapse NB samples were used to find somatic mutations. In NB cell lines, silencing by short hairpin RNA and transient overexpression of *FGFR1* were performed to evaluate the effect of the identified mutation by cell growth, invasion and cologenicity assays. HEK293, SHSY5Y and SKNBE2 were selected to investigate subcellular wild-type and mutated protein localization. FGFR1 inhibitor (AZD4547), alone or in combination with PI3K inhibitor (GDC0941), was used to rescue malignant phenotypes induced by overexpression of FGFR1 wild-type and mutated protein.

**Results:**

High *FGFR1* expression correlated with low relapse-free survival in two independent NB gene expression datasets. In addition, we found the somatic mutation N546K, the most recurrent point mutation of *FGFR1* in all cancers and already reported in NB, in one out of 19 matched primary and recurrent tumors. Loss of *FGFR1* function attenuated invasion and cologenicity in NB cells, whereas *FGFR1* overexpression enhanced oncogenicity. The overexpression of FGFR1^N546K^ protein showed a higher nuclear localization compared to wild-type protein and increased cellular invasion and cologenicity. Moreover, N546K mutation caused the failure in response to treatment with FGFR1 inhibitor by activation of ERK, STAT3 and AKT pathways. The combination of FGFR1 and PI3K pathway inhibitors was effective in reducing the invasive and colonigenic ability of cells overexpressing FGFR1 mutated protein.

**Conclusions:**

*FGFR1* is an actionable driver oncogene in NB and a promising therapy may consist in targeting *FGFR1* mutations in patients with therapy-resistant NB.

**Supplementary information:**

The online version contains supplementary material available at 10.1186/s12935-022-02587-x.

## Background

Neuroblastoma (NB) arises from malignant transformation of neural crest-derived precursors of the peripheral sympathetic nervous system and occurs in 5% of pediatric cancers in patients younger than 19 years [1]. The discovery of genomic markers such as *MYCN* amplification, 17q gain, 11q and 1p36 deletions has greatly improved risk stratification and prognosis of younger affected patients [2]. Instead, different genomic aberrations characterize NB in late childhood and adolescence, often showing 19p loss and 1q gain [3]. Additionally, genome-wide association studies (GWAS) [4] and candidate gene approaches [5–10] have identified multiple DNA polymorphisms influencing NB susceptibility and clinical phenotype that may represent novel potential outcome predictors [11, 12]. High-risk NBs comprise nearly half of all NBs and have a long-term survival of < 50%, with almost 60% of affected children being non-responsive to advanced treatments and dying due to relapse [13]. Although nowadays novel biomarkers such as microRNAs have been identified as powerful tools in diagnosis and prognosis for patients with NB [14, 15], high-risk disease treatment remains challenging. More recently, it has been shown that, among high-risk, gene expression-based signatures can identify children with higher risk disease who would benefit from new and more aggressive therapeutic approaches [16, 17]. Next generation sequencing studies have documented a paucity of mutations in recurrently affected genes in primary NB and an increase of “potentially actionable” mutations in relapse [18–20]. In primary tumors, mutations in *ALK*, *ATRX* and *TERT* have been identified as the most frequent genetic abnormalities [21–23], whereas in relapse an increased number of damaging or deleterious mutations in cell motility and cell survival pathways (e.g. PI3K/AKT/mTOR, MAPK or noncanonical Wnt pathways) has been reported [24]. Moreover, the selection of subclones with driver mutations in the RAS-MAPK pathway between the primary and the relapse tumors may occur as resistance mechanisms [19], but more research is needed to unravel the underlying causes. These data suggest that NB undergoes substantial mutational evolution during therapy and that relapsed disease is more likely to be driven by a targetable oncogenic pathway. Recently, we reported that somatic noncoding variants located in regulatory DNA elements specifically active in NB tumors can contribute to tumorigenesis [25].

Fibroblast growth factor (FGF) signaling cascades throught FGF receptor 1 (FGFR1) leads to the activation of MAP kinases. Alterations in FGFR1 have been reported in 3.63% of all cancers, with breast carcinoma, non-small cell lung carcinoma, colorectal adenocarcinoma, malignant glioma and ovarian neoplasms showing the greatest prevalence of abnormalities [26]. The most common alterations in *FGFR1* are amplifications (2.34%), point mutations (1.20%) and gene loss (0.33%) [26]. Among the point mutations, the most recurrent one is N546K (0.14%) [26], that has been found in primary NB [18, 27] and in the paired relapsed tumors [18, 19]. Moreover, in addition to the already reported relapsed NB case [18], N546K mutation has also been recently reported in 6 patients [28]. Specifically, N546K represents an activating mutation that alters *FGFR1* auto-phosphorylation [29], resulting in an increase of kinase activity and malignant transformation in Ewing sarcoma and brain tumors [30–34].

FGFR constitutes a promising druggable target in cancer and different approaches for inhibiting FGFR, including selective and nonselective FGFR small-molecule tyrosine kinase inhibitors (TKIs), monoclonal antibodies against FGFRs and FGF ligand traps are under investigation in several phase I/II clinical trials [35].

The aim of this study was to characterize *FGFR1* as NB cancer-driver gene and to evaluate its role as therapeutic target with in vitro studies.

## Methods

### Microarray-KAPLAN SCAN

R2 web tool [36] was used to predict the association of *FGFR1* expression with survival of NB patients. In brief, for each gene, R2 calculates the optimal cut-off in the expression level to divide patients in ‘good’ and ‘bad’ prognosis cohorts. Samples within a dataset are sorted based on the expression of the investigated gene and are divided in two groups. All the cut-off expression levels and their resulting groups are analyzed related to patient survival. For each cut-off level and grouping, the log-rank significance of the projected survival is calculated. The best probability value (*p*-value) and the corresponding cut-off are selected. The cut-off level is reported and was used to generate the Kaplan–Meier curves. These depict the log-rank significance (raw *p*) as well as the *p*-value corrected for multiple testing (Bonferroni correction) of the cut-off levels for each gene. Kaplan scan analysis was performed to estimate the overall and relapse-free survival related to *FGFR1* expression in the following microarray datasets: Seeger dataset (102 International NB Staging System stage 4 patients without *MYCN* amplification), Versteeg dataset (88 patients with different clinical characteristics) and Asgharzadeh TARGET dataset (247 patients).

### Whole genome sequencing

In-house Wholegenome sequencing (WGS) data:WGS of 10 normal-primary-relapse NB sample triplets was performed on an Illumina HiSeq1500 platform. The paired-end sequencing produced 150 bp long reads. Alignment files were obtained by mapping reads versus GRCh37/hg19 reference genome assembly. Somatic single nucleotide variants (SNVs) and insertions and deletions (INDELs) were detected with MuTect [37] and Strelka [38], respectively.

Publicly available WGS data (Target): we obtained access to WGS of NB from the TARGET project [39] (Accession: phs000218.v21.p7; Project ID: #14831) and included, in our analysis, 9 normal-primary-relapse NBs for which somatic variants were available. The functional annotation of somatic variant calls was performed with ANNOVAR [40] and FunSeq2 [41].

### Copy number variation analysis

We evaluated the copy number (CN) status of *FGFR1* in NB patients of the TARGET-NB project. Open access level 3 (L3) copy number segmentation data of 381 NB samples [42] were downloaded from NIH Office of Cancer Genomics website [43]. The R-Bioconductor “copynumber” package [44] was implemented to estimate CN status starting from Log R Ratio (LRR) and B Allele Frequency (BAF) information. For both datasets, we set stringent cutoffs to call CN changes: CN losses were called for LRR below − 0.42 (CN < 1.5); normal LRR values were between − 0.42 and 0.58 (CN ranging from 1.5 to 3); CN gains were called if LRR was between 0.58 and 1.3 (CN ranging from 3 to 4.9); we called amplification for LRR greater than or equal to 1.3 (CN ≥ 4.9). RefSeq *FGFR1* transcript variant 1 (NM_023110) genomic coordinates were taken from UCSC genome browser [45] and used to search for the presence of CN variants (CNVs) in samples of the above mentioned datasets.

### Cell culture

The human SHSY5Y and HEK293 cells were grown in Dulbecco’s modified Eagle’s medium (DMEM); SKNBE2 cells were grown in DMEM/Nutrient Mixture F-12 (F-12). Both cell lines were supplemented with 10% heat-inactivated fetal bovine serum (FBS) (Sigma), 1 mM l-glutamine, penicillin (100 U/ml) and streptomycin (100 µg/ml) (Invitrogen), and cultured at 37 °C, under 5% CO_2_ in a humidified atmosphere. AZD4547 and GDC0941 were diluted in dimethyl sulfoxide (DMSO) at 10 mM/ml and stored at − 20 °C until use. The inhibitors were diluted to 0.1 µM and 1 µM in culture medium without serum.

### Production of lentiviral particles and infection of cell lines

To knock-down FGFR1 expression, the GIPZ lentiviral shRNAmir that targets human *FGFR1* were purchased from Open Biosystems (Thermo Fisher Scientific, Inc.). We used two different short hairpin RNAs (shRNAs) for *FGFR1* gene. The shRNAs against *FGFR1* were shFGFR1#A (V3LHS_644622) and shFGFR1#B (V3LHS_634642). A non-silencing GIPZ lentiviral shRNAmir was used as control (RHS4346). HEK293T were transfected using 10 µg shRNA plasmid DNA, 30 µl Trans-Lentiviral Packaging Mix (OpenBiosystem), and 25 µl TransFectin (BioRad), in 10-mm plates. The supernatants (10 ml per condition) were harvested after 24 h, centrifuged at low speed to remove cell debris, and filtered through 0.45-µm filters. Cells transduction was performed as previously described [46].

### Western blotting

Cell pellets were resuspended and lysed in RIPA buffer (50 mM Tris-HCl, pH 7.5, 150 mM NaCl, 1% Triton X-100, 10% glycerol), complemented with protease and phosphatase inhibitors cocktail (ThermoScientific). Total proteins extracts concentrations were determined through Bradford assay (Bio-Rad). Cytosol and nucleus protein fractions were obtained as previously described [47].

After 1 h blocking with 5% non-fat dried milk (EuroClone) or bovine serum albumin (SERVA) in Tris-buffered saline with 0.1% Tween (TBS-T), membranes were incubated with primary antibodies at 4 °C overnight. Primary antibodies used: anti-pFGFR1 (06-1433, Millipore), anti-FGFR1 (Abcam ab137765), anti-pSTAT3 (D3A7, Cell Signaling), anti-STAT3 (06596, Millipore), anti-pAKT1 (ab81283; Abcam), anti-AKT1 (ab32505; Abcam), anti-pERK1/2 (ab32538; Abcam), anti-ERK1/2 (ab17942; Abcam), anti t-GFP (TA150041, Origene) and β-actin (Sigma, A5441). After membrane incubation with horseradish-peroxidase-conjugated anti-rabbit secondary antibody (Immuno Reagents), the positive bands were visualized using the ECL kit SuperSignalTM West Pico PLUS Chemiluminescent Substrate (Thermo Scientific) as previously shown [48].

### Real-time PCR

Total RNA extraction using TRIzol LS Reagent (Invitrogen) and cDNA retrotranscription using the High-Capacity cDNA Reverse Transcription Script (Applied Biosistem) was performed according to the manufacturer protocol. Specific primers for *FGFR1* (Forward: 5′-GCTAAAGCACATCGAGGTGAATG-3′; Reverse: 5′-TCTCTTTGTCGGTATTAACTCC-3’) and *β-Actin* (Forward: 5′-CGTGCTGCTGACCGAGG-3′; Reverse: 5′-GAAGGTCTCAAACATGATCTGGGT-3′) were designed by PRIMEREXPRESS software (Applied Biosystems). Real-time PCR (RT-PCR) was performed using SYBR Green PCR Master Mix (AppliedBiosystems) in the 7900HT Fast Real-Time PCR System (Applied Biosystems). The experiments were carried out in triplicate for each data point. Relative gene expression was obtained using the 2−ΔCT method, where the ΔCT was calculated using the differences in the mean CT between the selected genes and the internal control (β-actin).

### Cell viability assay

Cells were seeded as six replicates into 96-well plates at a density of 10 × 10^3^ cells per well. Cell viability was measured by evaluating metabolic conversion (by viable cells) of the dye 3-(4,5-dimethylthiazol-2L)-2,5-diphenyltetrazolium bromide (MTT), according to the manufacturer protocol (Promega) as previously described [46]. For drugs treatments, different drugs concentrations were added to culture medium and cell viability was measured after 24 h, 48 h and 72 h. Inhibitory concentration (IC_50_) values were calculated using nonlinear best fit regression analysis by Excel.

### Invasion assay

Transwell chambers (Corning) were pre-coated with matrigel (BD Biosciences) at 37 °C for 30 min. 80,000 cells resuspended in 350 µl serum-free medium were added to the upper compartment, and 750 µl DMEM containing 10% FBS, (Sigma), 1 mM l-glutamine, penicillin (100 U/ml) and streptomycin (100 µg/ml) (Invitrogen) was added to the lower chamber. Then, cells were incubated for 24 h at 37 °C with 5% CO_2_. Transwell chambers were removed from the 24-plate and migrated cells were stained as previously described [49]. The invading cells were counted using the LeicaApplicationSuite/AF software and DMI4000B microscope (Leica Mycrosystem). Chamber photos were acquired with 10× objective.

### Colony formation assay in soft agar

The colony formation assay was performed to analyze anchorage-independent cell growth. 200,000 cells were plated in 0.35% agar on a bottom layer of 1% agar in the 35-mm dishes of 6-well plates (Corning). The plates were incubated at 37 °C for 4 weeks, and then stained with 0.01% crystal violet. Colonies with 20 cells or more were counted using the LeicaApplicationSuite/AF software and DMI4000B microscope (Leica Mycrosystem) with 10× objective. Means and standard deviations were calculated from three independent experiments.

### Site-directed mutagenesis

Site-directed mutagenesis was performed on plasmid containing the coding sequence of human *FGFR1* (RG202080, Origene) using a PCR-based strategy through KAPA HiFi Hot Start DNA polymerase (KAPA BIOSYSTEMS, London, United Kingdom). To introduce *FGFR1* missense mutation N546K, primers were designed by the online tool Primer-BLAST. Reaction mixture contained 0.5 U KAPA HiFi HotStart DNA Polymerase, 300 µM KAPA dNTP Mix, 0.3 µM forward and reverse primers, 1× KAPA HiFi Fidelity Buffer and 50 ng plasmid DNA as template. Ultimately, PCR reaction was performed in the following conditions: 95 °C for 5 min; 35 cycles of 98 °C for 20 s, 66 °C for 15 s and 72 °C for 90 s; and finally 72 °C for 5 min. Product was treated with 1 U DpnI (NEB, USA) for 1 h at 37 °C, and heat-inactivated at 80 °C for 20 min. The new vector was analyzed by electrophoresis on 0.8% agarose gel and sequenced for validation.

### Cell transfection

HEK293, SHSY5Y and SKNBE2 cells were seeded at a density of 250,000 cells per well in a 6-well plate and transfected with 2.5 µg of pCMV6 empty vector or pCMV6 expressing FGFR1^wt^ protein or pCMV6 expressing FGFR1^N546K^ protein and 3 µl of TransFectin™ Lipid Reagent (Bio-Rad). Transiently transfected cells were subsequently starved in serum-free medium for 4 h and were harvested after 48 h.

### ImageStream^X^ Mark II Flow Cytometer acquisition and data analysis

Cells were fixed in 4% paraformaldehyde (10 min), permeated with 0.2% Triton X-100 (15 min), and blocked with 1% bovine serum albumin (30 min). The anti-FGFR1 primary antibody (ab824, Abcam) was incubated for 90 min, and the AlexaFluor 647 goat anti-rabbit (A27040, Invitrogen) secondary antibody for 45 min. Then, cells were incubated with DAPI (Sigma) for 10 min to stain nuclei. A filter of 30-µm was used to remove cell aggregates. ImageStream^X^ Mark II Flow Cytometer (EMD Millipore) was used to acquire single cells images at 60× magnification. The acquired raw image file (.rif) contained among 500 and 2000 events (10–30 events per second). The analysis of single cells fluorescence intensity and nucleus diameter was performed by using IDEAS software (version 6.2.64.0). To consider only single cells, a dot plot showing area versus aspect ratio (AR) was created. To estimate FGFR1 intensity in nuclear region, we generated a morphology mask that defined nucleus stained by DAPI and to measure the fluorescent signal in nuclear area.

### Immunofluorescence confocal microscopy

After 48 h from transfection, HEK293, SHSY5Y and SKNBE2 cells were seeded on polilysine coated glass coverslips (Microtech S.R.L) overnight. Coverslips were fixed in 4% paraformaldehyde (10 min), permeated with 0.2% Triton-100 (15 min) and then blocked in 1% bovine serum albumin (30 min). The anti-FGFR1 primary antibody (ab824) was incubated for 90 min. Coverslips were then incubated in goat anti-Mouse IgG (H+L) secondary antibody Alexa Fluor 546 (Invitrogen A-11030) for 45 min. Coverslips were then stained with DAPI (Sigma) for 10 min. Slides were mounted with Mowiol® 4-88 (Sigma-Aldrich, 81381) and visualized using a Leica TCS SP8 STED 3× confocal microscope (Leica Microsystems CMS GmbH).

### Neurospheres assay

Neurospheres formation assay was performed in serum-free medium containing half mixture of F-12 and DMEM Low Glucose, supplemented with 20 ng/ml Epidermal growth factor (EGF), 40 ng/ml Basic FGF (bFGF), 2% serum-free medium supplement B-27 (Gibco, ThermoFisher Scientific) and 1% l-glutamine/penicillin–streptomycin. Cells were seeded in the 35-mm dishes of 6-well plates (Corning). Plates were incubated at 37 °C for 3 days following the cells seeding in serum-free medium. Spheres were observed and acquired through LeicaApplicationSuite/AF software and DMI4000B microscope (Leica Mycrosystem). Chamber photos were acquired with 10× objective.

### Statistical analysis

The differences among groups were analyzed using unpaired student’s *t*-test. *p*-value < 0.05 were considered statistically significant. *p-value ≤ 0.05, **p-value ≤ 0.01, ***p-value ≤ 0.001.

## Results

### *FGFR1 *expression is associated with bad clinical outcomes in NB patients

**Fig. 1:**
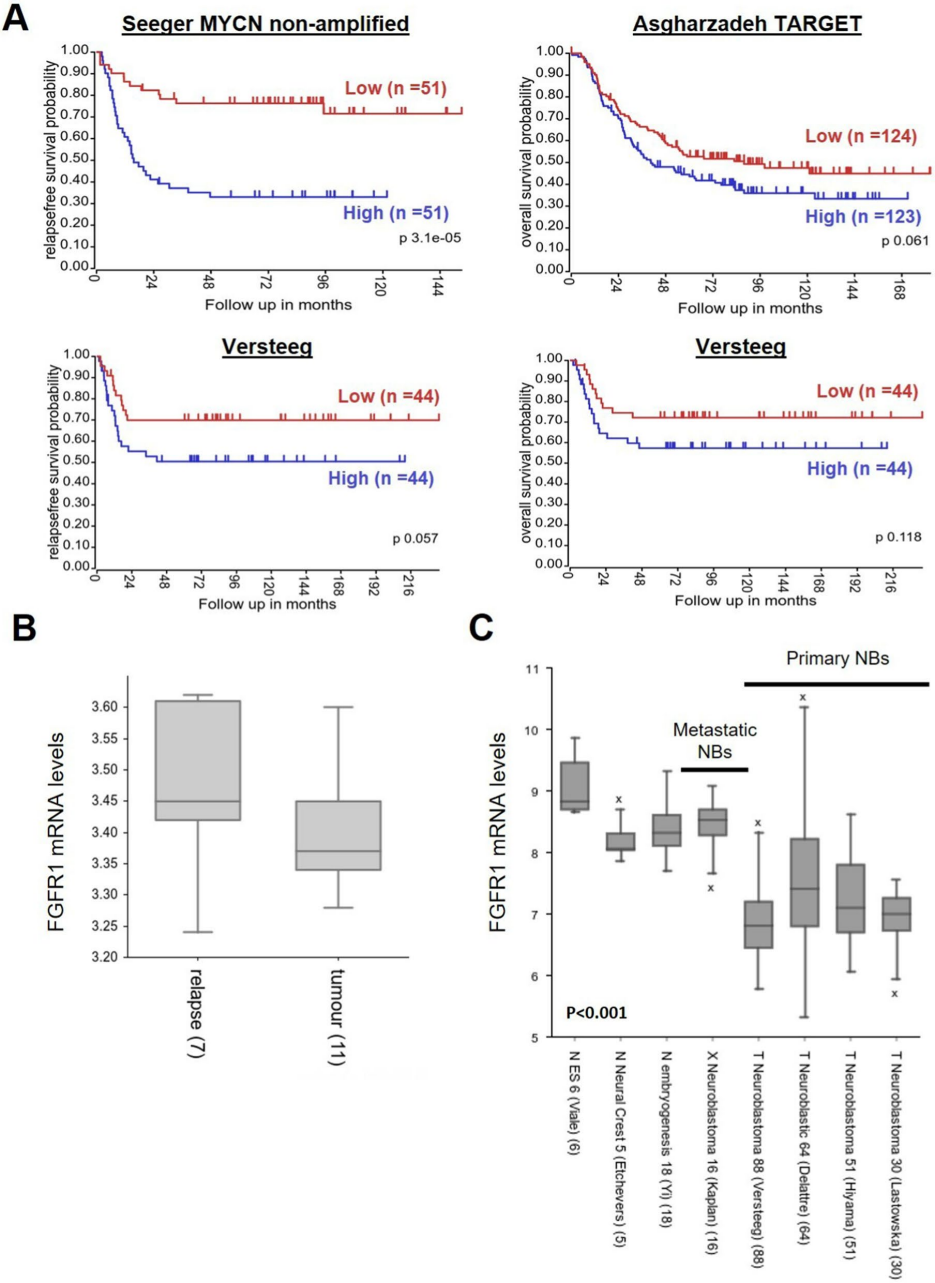
Association of *FGFR1* expression with clinical outcomes in patients with NB. **A** The association of *FGFR1* expression with clinical outcomes was evaluated in the following datasets: Seeger, Asgharzadeh TARGET and Versteeg. (n = number of patients). **B** *FGFR1* expression analysis in datasets of primary and relapsed tumors. **C ***FGFR1* expression levels in two embryonic cells (ES), one neuronal crest cells (NC), one metastatic xenograft tumors (X) and four primary NB (T) datasets. In **B** and **C** the number of samples is reported in brackets. p = *p*-value

The association of *FGFR1* expression with clinical outcomes was evaluated in three datasets deposited in R2 microarray web tool [36]: Seeger dataset (102 patients with high-risk NB); Versteeg dataset (88 patients) and Asgharzadeh TARGET dataset (247 patients). Kaplan–Meier analysis showed that higher *FGFR1* expression was significantly associated with inferior relapse-free survival in Seeger dataset (*p*-value = 3.1 × 10^−5^) and in Versteeg dataset (*p*-value = 0.057). In contrast, correlation of *FGFR1* with overall survival was not significant in Asgharzadeh TARGET dataset (*p*-value = 0.061) and in Versteeg dataset (*p*-value = 0.118) (Fig. 1A).

*FGFR1* expression analysis in a dataset of 11 primary and 7 relapsed tumors showed a higher *FGFR1* expression in relapsed NB samples without reaching the significance level (*p*-value = 0.28), probably due to the limited number of samples (Fig. 1B).

Finally, we observed that *FGFR1* mRNA levels in metastatic xenograft tumors were higher than those of NB primary tumors (*p-*value < 0.001) but were comparable to those of embryonic cells and neuronal crest cells (Fig. 1C).

### *FGFR1* somatic mutations and copy number variations in NB patients

We analyzed WGS data at *FGFR1* locus (including 50 kb surrounding regions) from 19 paired diagnostic and relapse NBs. WGS data from 10 samples were obtained in our laboratory whereas 9 were downloaded from TARGET project repository.

We found the hotspot mutation N546K in *FGFR1* in one tumor at diagnosis and relapse (Table 1). No other putative coding pathogenic mutations were found. We also investigated potential pathogenic function of noncoding point mutations. To this purpose, we annotated each mutation with DNase I hypersensitive sites, known to define active regulatory DNA elements, in SKNSH NB cells (ENCODE data). No potential pathogenic mutations located in DNA regulatory sites were found (Table 1).

Since *FGFR1* amplifications have been associated with other cancers, we analyzed copy number variations in a public dataset of 381 NBs. No significant amplification of *FGFR1* was found (Additional file 1: Fig. S1).

**Table 1:** Coding and noncoding somatic mutations found at FGFR1 locus in 21 primary-relapse pairs NB tumors analyzed by whole genome sequencing

Sample ID	Type	Position/change	Location	Gene	Amino acid change	Band	CADD score	COSMIC ID	SNP ID	ENCODE annotation
TR008	R	chr8:38227325:G>T	Intronic	WHSC1L1	–	8p11.23	2.48	–	–	Enhancer^b^
TR008	R	chr8:38236248:C>G	Intronic	WHSC1L1	–	8p11.23	1.78	–	–	–
TR008	P	chr8:38246999:C>G	Intronic	LETM2	–	8p11.23	1.77	–	–	–
TR008	R	chr8:38246999:C>G	Intronic	LETM2	–	8p11.23	1.77	–	–	–
TR001	P	chr8:38267939:A>C	Downstream	FGFR1, LETM2	–	8p11.23	1.03	–	–	TFP(CTCF)
SP_2_T	P	chr8:38268616:C>A	Downstream	FGFR1	–	8p11.23	3.58	–	–	TFP(CTCF)
TR003	R	chr8:38273003:A>T	Intronic	FGFR1	–	8p11.23	2.25	–	–	–
PATNKP	P	chr8:38274849:G>T	Exonic	FGFR1	N457K	8p11.23	29.70	Yes^a^	rs779707422	–
PATNKP	R	chr8:38274849:G>T	Exonic	FGFR1	N457K	8p11.23	29.70	Yes^a^	rs779707422	–
TR007	P	chr8:38282676:A>T	Intronic	FGFR1	–	8p11.23	2.96	–	–	DHS(MCV-1)
TR001	P	chr8:38288403:G>C	Intronic	FGFR1	–	8p11.23	0.22	–	–	DHS(MCV-2); TFP(SMARCC1)
TR008	P	chr8:38295809:T>C	Intronic	FGFR1	–	8p11.23	0.23	–	rs975858205	–
PAUDDK	P	chr8:38296890:T> A	Intronic	FGFR1	–	8p11.23	17.63	–	–	–
TR003	P	chr8:38301604:T>G	Intronic	FGFR1	–	8p11.22	2.88	–	rs947373873	–
PATNKP	P	chr8:38311785:C>G	Intronic	FGFR1	–	8p11.22	2.90	–	–	
PATNKP	R	chr8:38311785:C>G	Intronic	FGFR1	–	8p11.22	2.90	–	–	–
TR008	R	chr8:38319864:G>A	Intronic	FGFR1	–	8p11.22	0.75	–	–	–
TR007	R	chr8:38324367:G>A	Intronic	FGFR1	–	8p11.22	9.06	–	–	Enhancer^b^; TFP(SIN3A, TAF7, TCF12, YY1)
TR008	P	chr8:38337889:A>G	Intergenic	FGFR1(dist=11537)	–	8p11.22	3.92	–	–	–
TR008	R	chr8:38338780:A>G	Intergenic	FGFR1(dist=12428)	–	8p11.22	18.68	–	–	DHS(MCV-2)
TR008	R	chr8:38338784:C>G	Intergenic	FGFR1(dist=12432)	–	8p11.22	17.75	–	rs201380585	DHS(MCV-2)
TR006	R	chr8:38349608:A>G	Intergenic	FGFR1(dist=23256)	–	8p11.22	0.61	–	–	–
TR006	R	chr8:38350422:C>G	Intergenic	FGFR1(dist=24070)	–	8p11.22	10.82	–	–	–
TR008	P	chr8:38350507:A>C	Intergenic	FGFR1(dist=24155)	–	8p11.22	0.03	–	–	–
TR006	P	chr8:38350642:T>C	Intergenic	FGFR1(dist=24290)	–	8p11.22	0.04	–	–	–
PATNKP	R	chr8:38357592:C>A	Intergenic	FGFR1(dist=31240)	–	8p11.22	4.03	–	–	–
TR006	R	chr8:38369276:A>C	UTR3	C8orf86	–	8p11.22	0.45	–	rs565928745	–

*P* primary, *R* relapse, *TFP* transcription factor binding peak, *DHS* DNase I hypersensitive sites, *MCV-1, MCV-2* cell lines of the ENCODE catalog

^a^ID = COSM3670398, COSM1284966, COSM1284968, COSM1284967, COSM19176; OCCURENCE = 5(central_nervous_system), 1(autonomic_ganglia)

^b^Segway/ChromHMM-predicted enhancer

### *FGFR1* silencing impairs cell growth, invasion and colonigenicity in NB cells

We investigated the role of *FGFR1* in two NB cell lines: SHSY5Y *MYCN* non*-*amplified and SKNBE2 *MYCN-*amplified cells.

**Fig. 2:**
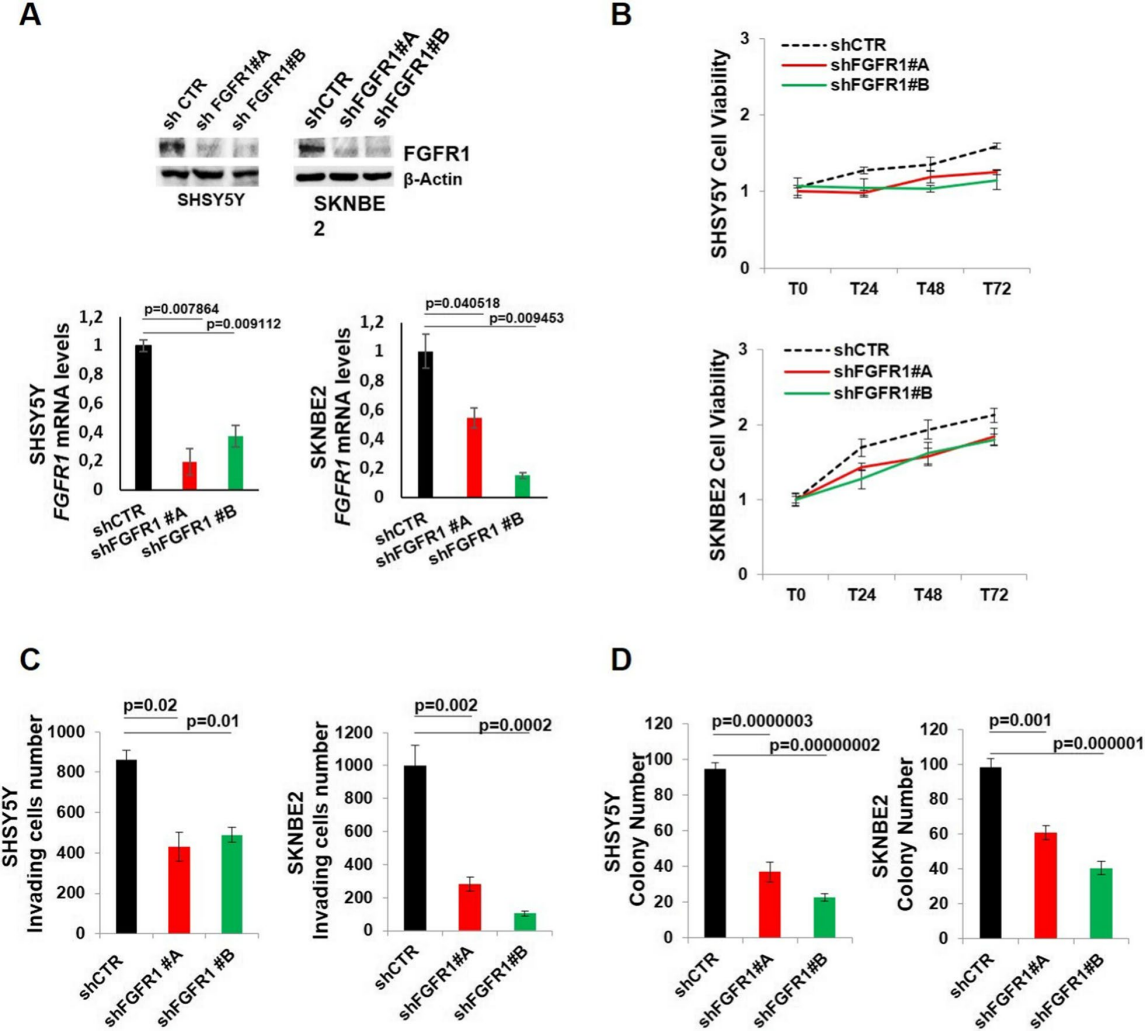
*FGFR1* silencing impairs cell growth, cell invasion and clonogenicity in NB cells. **A** *FGFR1* silencing efficiency was evaluated by western blotting and RT-PCR in SHSY5Y and SKNBE2 transduced by lentiviral vectors encoding shFGFR1#A and shFGFR1#B. FGFR1 levels folded on shCTR mRNA levels are reported. **B** Cell viability in shFGFR1#A and shFGFR1#B cells is shown as fold change compared to shCTR. **C** Invading cells and **D** colony number in *FGFR1* silenced and control cells are reported. Vehicle = DMSO. p = *p*-value

We transduced SHSY5Y and SKNBE2 cells by lentiviral vectors encoding two independent shRNAs targeting FGFR1 (shFGFR1#A and shFGFR1#B) and a control shRNA (shCTR). Silencing efficiency was determined by western blotting and RT-PCR (Fig. 2A).

Cell viability of both SHSY5Y and SKNBE2 shFGFR1 (shFGFR1#A and shFGFR1#B) significantly decreased compared to cell viability of shCTR after 48 h and 72 h (*p*-value ≤ 0.05) (Fig. 2B), suggesting that *FGFR1* silencing impaired NB cell proliferation and cell growth.

Similarly, *FGFR1* silencing affected NB cell ability to migrate through a matrigel-coated membrane (Fig. 2C and Additional file 1: Fig. S2A) and the anchorage-independent growth, as shown by soft agar assay (Fig. 2D and Additional file 1: Fig. S2B). Hence, colony numbers and invading cell numbers in shFGFR1 cells significantly decreased compared to shCTR cells in both SHSY5Y and SKNBE2 cell lines.

### FGFR1^N546K^ exhibits a nuclear localization

FGFR1 is constitutively found in cell membrane, cytoplasm and nucleus [50]. Data samples contained in the Human Protein Atlas clearly show that FGFR1 can localize to the nucleus [51]. FGFR1 nuclear localization in three-dimensional model of breast cancer and pancreatic cancer can influence the expression of hundreds of genes and contribute to migratory phenotype [52–55]. Additionally, in embryonic stem cells (ESCs), FGFR1 nuclear localization may increase in developing brain cells during neuronal differentiation to Neuronal Progenitor Cells (NPCs) [53, 56].

In this study, we investigated FGFR1 localization in HEK293 cells and two NB cell lines, SHSY5Y and SKNBE2, overexpressing both FGFR1^wt^ and FGFR1^N546K^.

HEK293, SHSY5Y and SKNBE2 cells were transiently trasfected with pCMV6 expressing FGFR1^wt^ or FGFR1^N546K^ proteins and pCMV6 empty vector.

**Fig. 3:**
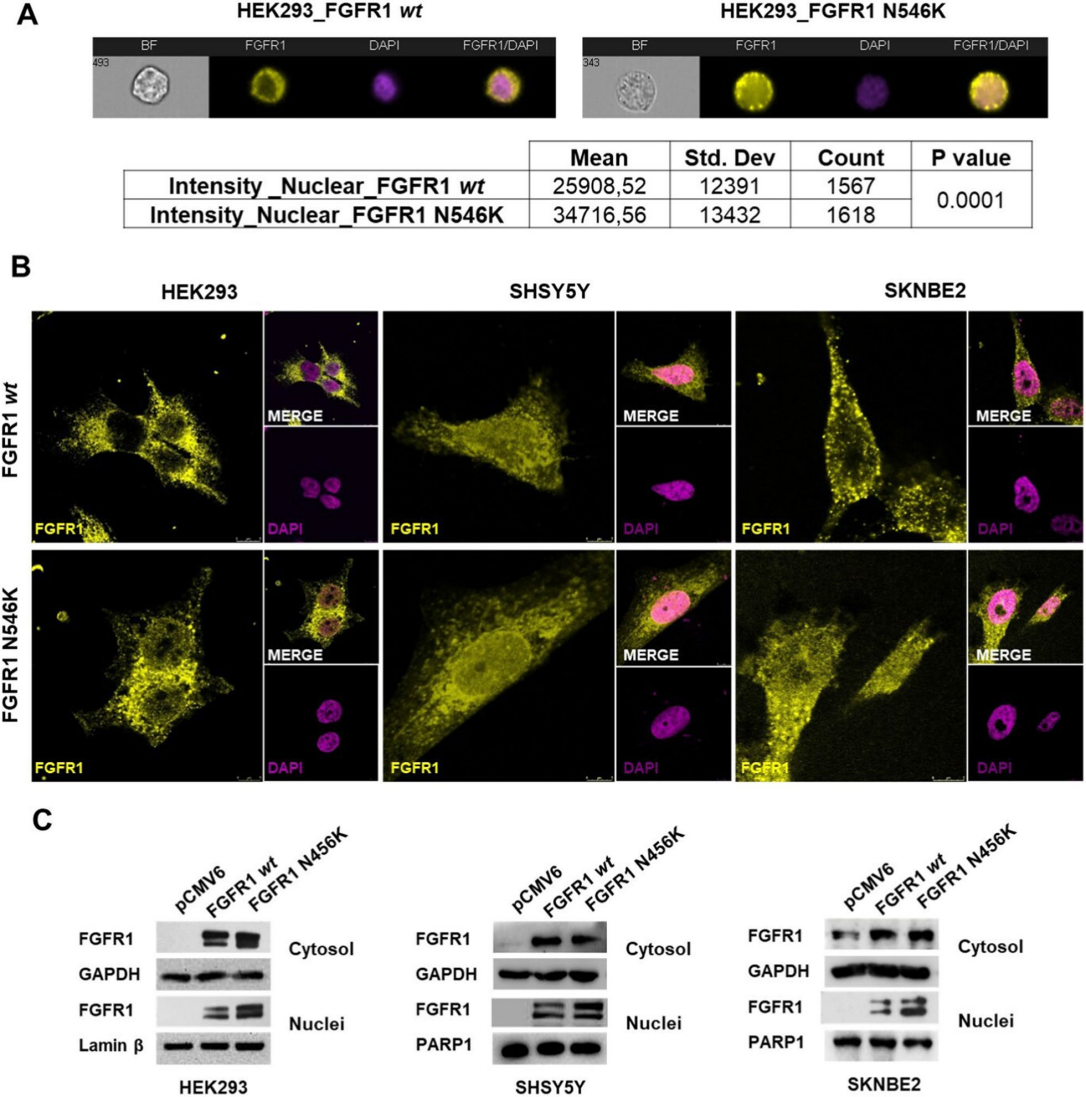
FGFR1^wt^ and FGFR1^N546K^ proteins localization. **A** FGFR1 localization in HEK293 overexspressing FGFR1^wt^ or FGFR1^N546K^ protein was analyzed by Image Stream flow Citometry. The representative figures of single cells at 60X magnification are shown above, whereas the values of mean intensity, standard deviation, cells counted and *p*-value are reported in the table. **B** Immunostaining of HEK293, SHSY5Y and SKNBE2 transfected with FGFR1^wt^ or FGFR1^N546K^ analyzed by confocal microscopy. **C** FGFR1 protein levels in both cytosol and nucleus protein fractions from HEK293, SHSY5Y and SKNBE2 transfected cells was evaluated by western blotting. GAPDH, Lamin β and PARP1 protein levels were used as loading controls

In HEK293, we examined FGFR1^wt^ and FGFR1^N546K^ proteins localization by ImageStream^X^ Mark II Flow Cytometer (Fig. 3A). FGFR1 nuclear signal intensity was calculated for 1000 HEK293 overexpressing FGFR1^wt^ and for 1000 HEK293 overexpressing FGFR1^N546K^ single cells. Abundant nuclear localization of FGFR1^N546K^ protein was statistically significant (p-value = 0.0001). This observation was confirmed by immunofluorescence confocal microscopy assay showing FGFR1^N546K^ protein mainly localized to nucleus, while FGFR1^wt^ protein mainly localized to cytosol (Fig. 3B).

In SHSY5Y and SKNBE2 cell lines we observed a higher nuclear localization of the protein in FGFR1^N546K^ overexpressing cells, compared to those overexpressing FGFR1^wt^ (Fig. 3B).

These data were validated by western blot analysis on cytosolic and nucleus fractions of HEK293, SHSY5Y and SKNBE2 transfected cells (Fig. 3C).

### FGFR1^N546K^ establishes crosstalk pathway activation and induces an increase in NB cellular invasion and colonigenicity

Early studies reported FGFR1^N546K^ mutation affects the conformational dynamics of the tyrosine kinase domain, resulting in gain-of-function and ligand-independent constitutive activation [29, 57, 58].

We performed western blotting analysis on total protein extracts from SHSY5Y and SKNBE2 transiently transfected with pCMV6 expressing FGFR1^wt^ or FGFR1^N546K^ proteins and pCMV6 empty vector to evaluate phosphorylated and total FGFR1, STAT3, ERK and AKT levels. The t-GFP protein level was used as transfection control and β-actin was used as loading control.

**Fig. 4:**
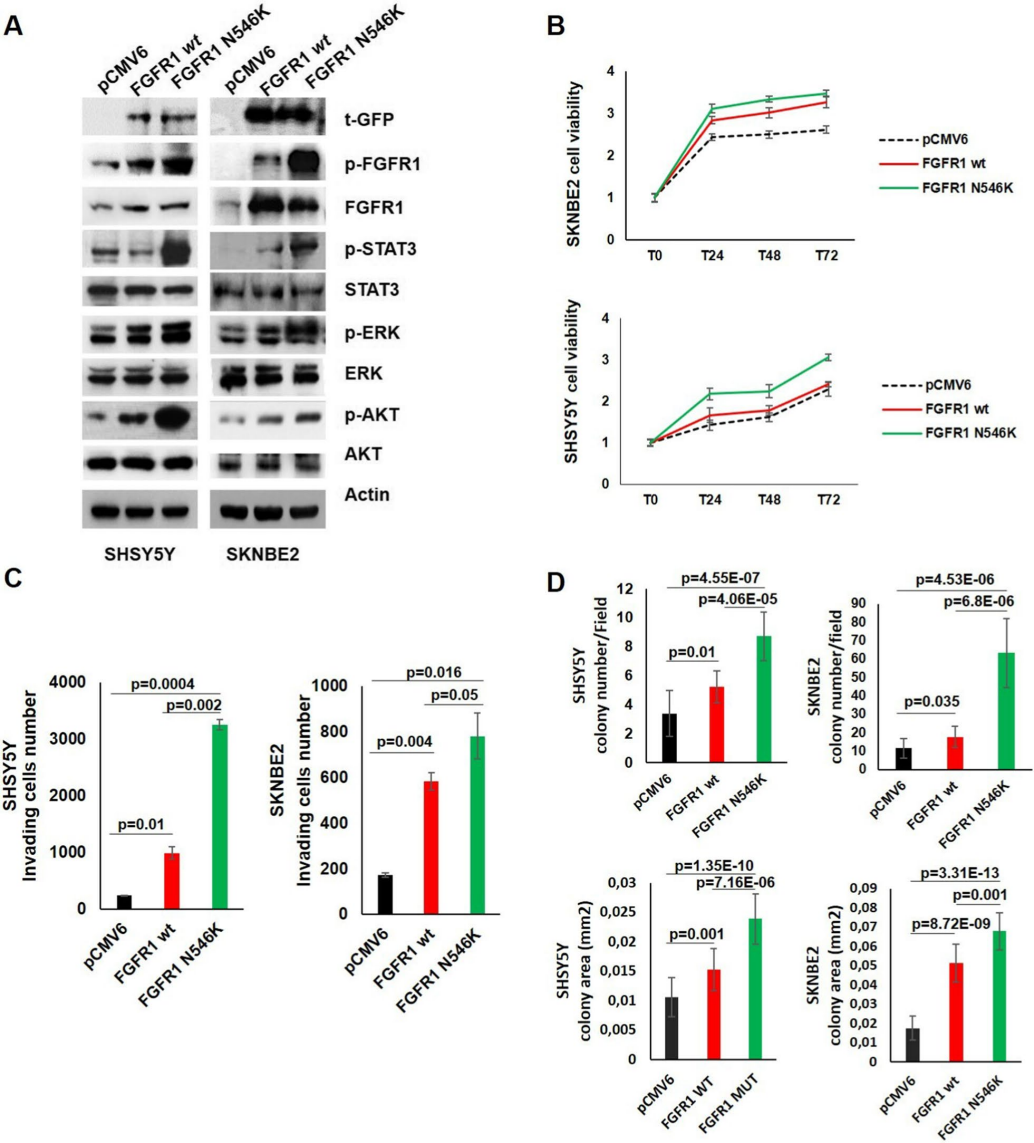
FGFR1^wt^ and FGFR1^N546K^ protein overexpression in NB cells. SH5YSY and SKNBE2 cells were transiently transfected with pCMV6-empty vector, pCMV6-FGFR1^wt^ and pCMV6-FGFR1^N546K^. **A** Total protein extracts were analyzed by western blotting to evaluate the levels of phosphorylated and total FGFR1, STAT3, ERK, and-AKT. The t-GFP and β-Actin protein levels were used as transfection control and loading control, respectively. **B** Cell viability in FGFR1^wt^ and FGFR1^N546K^ overexpressing cells is shown as fold change compared to the control (pCMV6). **C** Invading cells, **D** colony number and area were analyzed. mm^2^ = square millimetres. p = *p*-value

In NB cell lines, FGFR1^N546K^ overexpression enhanced the receptor kinase activity resulting in higher FGFR1 auto-phosphorylation. In addition, we observed a higher ERK, AKT and STAT3 phosphorylation in FGFR1^N546K^ compared to FGFR1^wt^ overexpressing cells (Fig. 4A).

We then evaluated cell viability in both SHSY5Y and SKNBE2 overexpressing FGFR1^wt^ and FGFR1^N546K^ protein compared to empty vector. Cell viability at 24 h, 48 h and 72 h significantly increased in FGFR1^wt^ and FGFR1^N546K^ overexpressing cells compared to pCMV6 empty vector (*p-*value ≤ 0.05) and FGFR1^N546K^ overexpressing cells had the highest cell viability (*p*-value ≤ 0.05) (Fig. 4B).

The ability of transiently transfected SHSY5Y and SKNBE2 cells to invade and migrate through a matrigel-coated membrane support was evaluated. The number of invading FGFR1^wt^ and FGFR1^N546K^ overexpressing cells increased significantly compared to control pCMV6 cells. Interestingly, the number of invading FGFR1^N546K^ overexpressing cells was even higher than the number of invading FGFR1^wt^ overexpressing cells (*p*-value ≤ 0.05) (Fig. 4C and Additional file 1: Fig. S2C).

In addition, we analyzed the capability of FGFR1^wt^ and FGFR1^N546K^ overexpressing cells to interfere with colonigenicity in SHSY5Y and SKNBE2 cell lines. FGFR1^wt^ overexpression resulted in an increase of colony number and colony area compared to the empty vector in both cell lines (Fig. 4D and Additional file 1: Fig. S2D). Moreover, FGFR1^N546K^ overexpression was associated with the highest colony number and colony area in both cell lines (Fig. 4D and Additional file 1: Fig. S2D).

### N546K FGFR1 mutation may confer resistance to AZD4547 treatment in NB cell lines

Since AZD4547 represents a small molecule inhibitor targeting FGFR1 aberrant activation currently used in clinical trial [59, 60], we investigated the effects of this drug on FGFR1^wt^ and FGFR1^N546K^ in SHSY5Y and SKNBE2 cells.

Firstly, we evaluated AZD4547 potency against pCMV6-empty vector, FGFR1^wt^ and FGFR1^N546K^ overespressing cells (Additional file 1: Fig. S3A).

We performed cell viability assays in both cell lines testing different AZD4547 concentrations (0.01 µM, 0.1 µM, 1 µM and 10 µM), and then we calculated the half maximal IC_50_ for this drug, which resulted comparable in SHSY5Y and SKNBE2 (Additional file 1: Fig. S3A).

Based on the IC_50_ results, we selected the lower concentration of AZD4547 (0.1 µM) able to decrease viability up to 20% in both cell lines (Additional file 1: Fig. S3A). Specifically, we decided to not test 1 µM AZD4547 because the treatment with this concentration showed 31% reduction in cell viability in SKNBE2 pCMV6-empty vector compared to vehicle cells (DMSO) (Additional file 1: Fig. S3A).

To investigate the early effect of the drug treatment on the inhibition of downstream pathways, cells overexpressing FGFR1^wt^ and FGFR1^N546K^ were incubated for 2 h in serum-free medium in presence of AZD4547 (0.1 µM) or vehicle (DMSO).

**Fig. 5:**
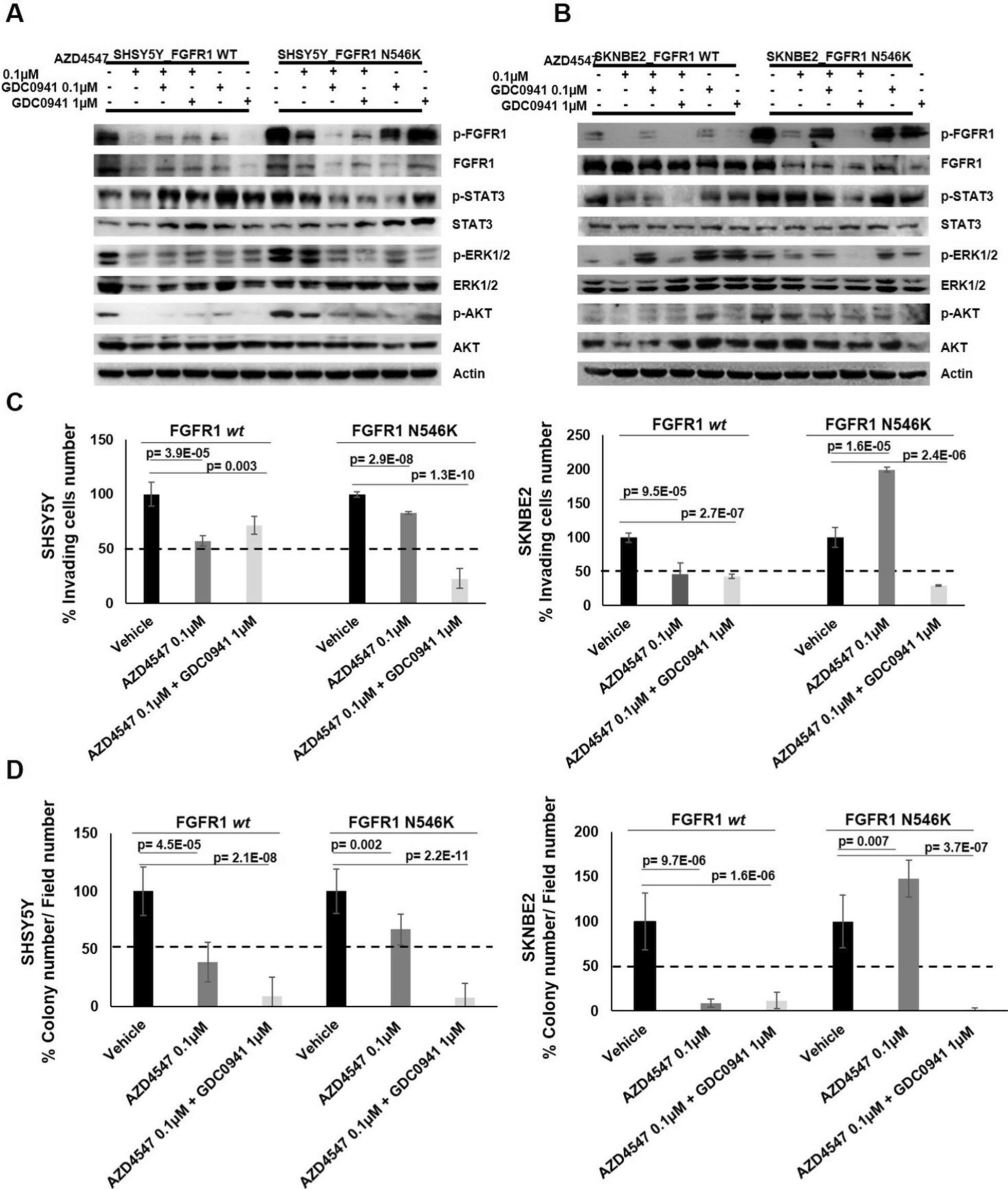
Targeting of FGFR1 signaling by combination treatment with AZD4547 and GDC0941. SH5YSY and SKNBE2 cells were transiently transfected with pCMV6-FGFR1^wt^, pCMV6-FGFR1^N546K^ and pCMV6 empty vector. **A**, **B** Total protein extracts were analyzed by western blotting to evaluate the levels of phosphorylated and total FGFR1, STAT3, ERK and AKT. The β-Actin protein levels were used as loading control. **C** The ability of cells treated with single AZD4547 0.1 µM and with combination of AZD4547 0.1 µM and GDC0941 1 µM to invade and migrate through a matrigel-coated membrane support was evaluated. The number of invading FGFR1^wt^ or FGFR1^N546K^ overexpressing cells are shown in percentage respect to untreated cells (100% vehicle). **D** The ability of cells to form neuropheres after treatment with AZD4547 0.1 µM alone and in combination with GDC0941 1 µM was evaluated. The colony number of FGFR1^wt^ or FGFR1^N546K^ overexpressing cells are shown in percentage respect to untreated cells (100% vehicle). Vehicle = DMSO; p = *p-*value

Total protein extracts were analyzed by western blotting and phosphorylation levels of FGFR1, STAT3, ERK and AKT were evaluated in relation to their respective total protein quotas. β-Actin protein levels were used as loading control (Fig. 5A, B).

In both cell lines overexpressing FGFR1^wt^, AZD4547 0.1 µM decreased phospho-FGFR1, phospho-ERK and phospho-AKT protein levels, while did not strongly decrease phospho-STAT3 protein levels (Fig. 5A, B).

In SHSY5Y overexpressing FGFR1^N546K^, AZD4547 did not show efficacy to decrease phospho-FGFR1, phospho-ERK, phospho-AKT and phospho-STAT3 protein levels, that remained abundant in cells (Fig. 5A). In SKNBE2 overexpressing FGFR1^N546K^, although AZD4547 0.1 µM decreased phospho-FGFR1 and phospho-ERK levels, phospho-AKT levels were not affected and phospho- STAT3 levels resulted even enhanced (Fig. 5B).

In line with western blotting results (Fig. 5A, B), AZD4547 0.1 µM treatment, by impairing FGFR1 signaling, led to a 50% reduction in invasive capacity (Fig. 5C and Additional file 1: Fig. S4A) and colony number (Fig. 5D and Additional file 1: Fig. S4B) in both SHSY5Y and SKNBE2 FGFR1^wt^ overexpressing cells compared to untreated cells.

In SHSY5Y FGFR1^N546K^ overexpressing cells AZD4547 0.1 µM treatment, that increased phospho-ERK levels and did not affect phospho-FGFR1 and phospho-AKT levels as previously shown (Fig. 5A), did not strongly impair cellular invasion (Fig. 5C and Additional file 1: Fig. S4A) and neurospheres formation capability (Fig. 5D and Additional file 1: Fig. S4B). On the other hand, we observed an increase in cellular invasion capacity (Fig. 5C and Additional file 1: Fig. S4A) and in colony number (Fig. 5D and Additional file 1: Fig. S4B**)** in SKNBE2 FGFR1^N546K^ overexpressing cells, probably due to STAT3 and AKT phosphorylation (Fig. 5B).

Altogheter, these data suggest that AZD4547 abolishes the pathway activation induced by FGFR1^wt^, but does not show a great effectiveness on those ehanced by FGFR1^N546K^. Hence, N546K mutation may establish a resistance to AZD4547 treatment through activation of AKT and STAT3 pathways.

### Targeting of FGFR1^N546K^ signaling by combination treatment with AZD4547 and GDC0941 decreases crosstalk pathways activation, invasion and neurosphere formation capability

Since AZD4547 alone resulted non-effective in the abolishment of FGFR1^N546K^ induced cross-pathways, we decided to use it in combination with GDC0941, a PI3K inhibitor already used in clinical trials [61, 62].

As previously done for AZD4547, we firstly tested different concentrations of GDC0941 (0.01 µM, 0.1 µM, 1 µM and 10 µM) alone in both cell lines transiently transfected with FGFR1^wt^ and FGFR1^N456K^ by performing cell viability assay (Additional file 1: Fig. S3B). Differently from AZD4547, GDC0941 IC_50_ was higher in SKNBE2 cells (Additional file 1: Fig. S3A, B).

Based on the IC_50_ results, we decided to test the combination of AZD4547 (0.1 µM) and GDC0941 (0.1 µM and 1 µM) on cell viability, and we selected the lower concentrations able to decrease viability up to 20% (Additional file 1: Fig. S3C). Particularly, we used two GDC0941 concentrations (0.1 µM and 1 µM) since GDC0941 has shown lower toxicity in SKNBE2 (Additional file 1: Fig. S3B, C).

To investigate the early effects of the combination treatment on the inhibition of downstream pathways, cells overexpressing FGFR1^wt^ and FGFR1^N546K^ were incubated for 2 h in serum-free medium in presence of AZD4547 (0.1 µM) and GDC0941 (0.1 µM and 1 µM) or vehicle (DMSO).

Our aim was to investigate if these combinations at low doses could be more effective than AZD4547 single treatment in NB cells overespressing FGFR1^N546K^.

The transfected cells were treated with single GDC0941 (0.1 µM or 1 µM) to test drug efficiency. In FGFR1^N546K^ overexpressing cells treated with GDC0941 alone, we observed a significant decrease only in phospho-AKT protein levels (Fig. 5A, B).

In cells overexpressing FGFR1^wt^, the combination treatment with AZD4547 (0.1 µM) and GDC0941 (0.1 µM or 1 µM) was not effective to decrease both phospho-STAT3 and phospho-ERK protein levels, which in contrast showed an increase probably due to a compensation mechanism following the inhibition of FGFR1 signaling (Fig. 5A, B). Of note, in both cell lines overexpressing FGFR1^N546K^, the combination of AZD4547 0.1 µM and GDC0941 1 µM showed the best in vitro efficacy for the inhibition of all the three examinated pathways, highlighted by the reduction of phosphorylated/total protein levels (Fig. 5A, B).

In SHSY5Y cells overexpressing FGFR1^wt^ protein, AZD4547 0.1 µM and GDC0941 1 µM combination, compared to AZD4547 single treatment, showed a lower reduction in cell invasion capability (Fig. 5C and Additional file 1: Fig. S4A) and a decrease of colony number higher than 50% (Fig. 5D and Additional file 1: Fig. S4B), probably due to increment of phospho-STAT3 and a strong decrease of phospho-AKT levels, respectively (Fig. 5A). In SKNBE2 overexpressing FGFR1^wt^, the combined and the AZD4547 single treatment showed a similar effect on cell invasion (Fig. 5C and Additional file 1: Fig. S4A) and colonigenic (Fig. 5D and Additional file 1: Fig. S4B) capacity, as result of similar downstream pathways activation (Fig. 5B). Interesting to note, in both FGFR1^N546K^ overexpressing cell lines treated with AZD4547 0.1 µM and GDC0941 1 µM we observed a reduction of over 50% of invasion and neurosphere capacity, as consequence of above mentioned downstream pathway impairments (Fig. 5A–C and Additional file 1: Fig. S4A, B).

Together, these results highlight that AZD4547 0.1 µM and GDC0941 1 µM combination treatment was able to decrease the activation of downstream pathways, cell invasion and neurosphere formation abilities enhanced by FGFR1^N546K^ overexpression in NB cells.

Therefore, AZD4547 and GDC0941 combination treatment may represent a promising therapeutic strategy to overcome the resistance mechanisms induced by FGFR1 N546K mutation under AZD4547 treatment alone.

## Discussion and conclusions

*FGFR1* is an emerging promising target for the treatment of adult cancers as breast, lung and gastric cancers with *FGFR1* amplification being the most common somatic alteration responsive to therapeutic intervention. However, although we found no *FGFR1* amplifications in NB samples, point mutations seemed to occur in primary and relapse tumors [19, 27, 28]. Here we re-analyzed the coding and noncoding DNA sequences of *FGFR1* gene in 19 matched primary and relapsed NB tumors and found the hotspot mutation N546K in one sample at diagnosis and relapse obtained by TARGET database repository, suggesting that this mutation undergoes clonal selection. Of note, a large sequencing study recently found the same mutation in 6 primary NB tumors and in one matched relapsed tumor sample [28]. *FGFR1* clone selection for rare resistant subclones has been also reported in lung and colorectal resistant tumors, thus revealing a change in variant allele frequency of *FGFR1* somatic variants [63, 64]. Of note, N546K mutation was also found in Ewing sarcoma and brain tumors [30–34].

Cancer process is thought to be triggered by the reactivation of embryonic mechanisms in stem cells of adult tissues, in an entirely inappropriate context [65]. In line with this observation, we have recently shown that altered expression of genes involved in embryogenesis, due to cancer risk genetic variants, may contribute to malignancy and metastasis in neural crest-derived tumors including NB [66].

FGFR1 activation in resistent- or advanced-tumors is consistent with an epithelial-to-mesenchymal transition (EMT) and FGFR1 nuclear localization [67–70]. Particularly, FGFR1 nuclear form is crucial for the expression of ESCs migration and neural crest formation genes and promotes also invasion and extracellular matrix changes in advanced pancreatic and breast cancer cells [55, 70]. Considering these previous findings, we hypothesize that *FGFR1* can act as a cancer-driver gene in NB and that mutations in this gene might activate embryonic signaling, therefore promoting the recurrence of the disease. Our *in vitro* data show that *FGFR1* silencing in NB cells impairs cell proliferation, cell invasion and cell growth and these effects are rescued in FGFR1^wt^ overexpressing cells. Accordingly, our gene expression analysis of different datasets showed that high *FGFR1* expression associated with metastatic and relapsed tumors and inferior relapse-free survival, suggesting its role in promoting disease progression and recurrence. Our data show that N546K mutation leads to nuclear localization of FGFR1 protein and to activation of downstream signaling (AKT and STAT3) which results in an increase of the invasive and colonigenic capacity of cells. Since FGFR1 can promote the activation of developmental genes in ESCs [71], we do not exclude that N546K may lead to a reactivation of embryonic signaling as a result of FGFR1 nuclear localization.

FGFR1 is a tyrosine kinase receptor that, once activated, phosphorylates specialized intracellular adapters upstream of MAPK1/2 signaling pathway and its inhibitors are broadly used in clinical trials for the treatment of breast, lung and gastric cancers with *FGFR1* amplification [59, 60, 72]. AZD4547 is a small molecule TKI able to inactivate FGFRs downstream signaling by occupying the ATP-binding pocket in the kinase domain [73]. It has been reported as one of the most effective compounds for FGFR1 signaling inhibition that can be used at low concentrations for the treatment of advanced tumors [74–76]. In FGFR1^wt^ overexpressing cells, we observed that AZD4547 treatment was sufficient to abrogate FGFR1 signaling through inhibition of phospho-FGFR1 and phospho-ERK activation, resulting in an impairement of invasion and colonigenic cell ability. On the other hand, in FGFR1^N546K^ overexpressing cells, treatment with AZD4547 alone lead to an increase of phospho-AKT and phospho-STAT3 levels. These findings further support that AZD4547 treatment, by targeting FGFR1, can induce resistance mechanisms [77–79].

Mostly, potential resistance mechanisms to FGFR1 inhibition can converge on de novo [80, 81] and/or re-activation [82] of several signaling cascades. In particular, the mechanisms of AZD4547 resistance involve gene fusion (JUDMID-BRAF), alternative pathways activation (RAS-MAPK, ErbB3/PI3K/AKT and MET pathways) and related molecular abnormalities (RASA1, PHLDA1, PTEN, STAT3) [77, 79]. As additional mutations or selection of clones present prior to treatment might activate resistance mechanisms, we hypothesize that therapeutic combination of FGFR1 and PI3K inhibitors may have a synergistic effect respect to FGFR1 inhibitor used alone. Several studies have shown that GDC0941, designed to bind the ATP-binding pocket of PI3K to prevent formation of phosphatidylinositol-3,4,5-triphosphate (PIP3), inhibits cell proliferation in vitro and in vivo [83, 84]. GDC0941 molecule is already used in clinical trials in combination with other drugs for the treatment of metastatic breast cancers [61, 62]. Here, we observed that the combination of AZD4547 and GDC0941 shows additive effect on malignant phenotypes in vitro by inhibiting STAT3, AKT and ERK signaling activated by FGFR1^N564K^ protein.

Taken together, our results suggest that *FGFR1* expression is crucial for NB progression. Preliminary findings further suggest that FGFR1^N546K^ overexpressing cells show a further increase in motility and a failure to respond to treatment with FGFR1 inhibitor by activating ERK, STAT3 and AKT pathways. These signaling cascades enhanced by N546K mutation can be suppressed using the combination of FGFR1 and its downstream pathways inhibitors.

Therefore, targeting *FGFR1* mutation may represent a promising clinical strategy for both preventing and overcoming acquired drug resistance and provide insights regarding potential precision medicine therapeutics to achieve the complete remission in high-risk NB.

## Supplementary Information

**Additional file 1: Figure S1.** FGFR1 CNVs in TARGET-NB samples. (A) Histogram reporting the distribution (y axis) of segmented Log R Ratio LRR values (x axis) for the cohort of 381 samples in the TARGET-NB project. Red vertical lines represent the cutoffs we used to call copy number (CN) changes. LOSS: LRR 0.58 (CN ranging from 1.5 to 3); GAIN: 0.58 ≥ LRR > 1.3 (CN ranging from 3 to 4.9); AMPLIFICATION: LRR ≥ 1.3 (CN≥4.9). (B) Bar plot showing the three CN categories we identified by using the thresholds described above. **Figure S2.** Representative images of (A) invasion and (B) soft-agar assays in silenced *FGFR1* SHSY5Y and SKNBE2 cells. (C) Invasion and (D) neurosphere assays in *FGFR1* overexpressing cells are shown. **Figure S3.** SH5YSY and SKNBE2 cells were transiently transfected with pCMV6- empty vector, pCMV6- -FGFR1wt, pCMV6- - FGFR1N546K.In these cell lines, MTT assay to determine the IC50 value of (A) AZD4547 and (B) GDC0941 to analyze their effect on cell viability (%). The IC50 value (that is, the concentration of drug which exhibited 50% cell) are reported in table under the corresponding graph. (C) Bar plot represented the cell viability (%) of described above cell lines treated with different concentrations of AZD4547 and GDC0941 combination. Vehicle=DMSO. * = p-value ≤ 0.05, ** = p-value ≤ 0.01, *** = p-value ≤ 0.001 **Figure S4. **Representative images of (A) invasion assay and (B) neurospheres assay in SHSY5Y and SKNBE2 overexpressing FGFR1^wt^ or FGFR1^N546K ^cells after treatment with AZD4547 0.1 µM alone and in combination with GDC0941 1 µM. Vehicle = DMSO.

## Data Availability

The datasets generated during and/or analyzed during the current study are available from the corresponding author on reasonable request.
