# Supplementary material for: FGFR1 is a potential therapeutic target in neuroblastoma
Source: Cancer Cell Int. 2022 Apr 29;22:174. doi: 10.1186/s12935-022-02587-x (PMC9052553; doi:10.1186/s12935-022-02587-x)
Supplement: Supplementary file 1 — Additional file 1: Figure S1. FGFR1 CNVs in TARGET-NB samples. (A) Histogram reporting the distribution (y axis) of segmented Log R Ratio LRR values (x axis) for the cohort of 381 samples in the TARGET-NB project. Red vertical lines represent the cutoffs we used to call copy number (CN) changes. LOSS: LRR 0.58 (CN ranging from 1.5 to 3); GAIN: 0.58 ≥ LRR > 1.3 (CN ranging from 3 to 4.9); AMPLIFICATION: LRR ≥ 1.3 (CN≥4.9). (B) Bar plot showing the three CN categories we identified by using the thresholds described above. Figure S2. Representative images of (A) invasion and (B) soft-agar assays in silenced FGFR1 SHSY5Y and SKNBE2 cells. (C) Invasion and (D) neurosphere assays in FGFR1 overexpressing cells are shown. Figure S3. SH5YSY and SKNBE2 cells were transiently transfected with pCMV6- empty vector, pCMV6- -FGFR1wt, pCMV6- - FGFR1N546K.In these cell lines, MTT assay to determine the IC50 value of (A) AZD4547 and (B) GDC0941 to analyze their effect on cell viability (%). The IC50 value (that is, the concentration of drug which exhibited 50% cell) are reported in table under the corresponding graph. (C) Bar plot represented the cell viability (%) of described above cell lines treated with different concentrations of AZD4547 and GDC0941 combination. Vehicle=DMSO. * = p-value ≤ 0.05, ** = p-value ≤ 0.01, *** = p-value ≤ 0.001 Figure S4. Representative images of (A) invasion assay and (B) neurospheres assay in SHSY5Y and SKNBE2 overexpressing FGFR1wt or FGFR1N546K cells after treatment with AZD4547 0.1 µM alone and in combination with GDC0941 1 µM. Vehicle = DMSO. [file 12935_2022_2587_MOESM1_ESM.docx]

**Additional file 1**

**
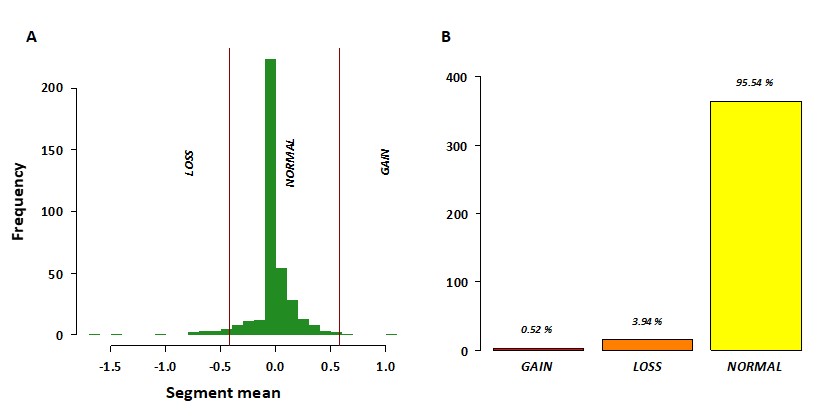
**

**Figure S1** FGFR1 CNVs in TARGET-NB samples. (A) Histogram reporting the distribution (y axis) of segmented Log R Ratio LRR values (x axis) for the cohort of 381 samples in the TARGET-NB project. Red vertical lines represent the cutoffs we used to call copy number (CN) changes. LOSS: LRR < -0.42(CN<1.5); NORMAL: -0.42 ≥ LRR > 0.58 (CN ranging from 1.5 to 3); GAIN: 0.58 ≥ LRR > 1.3 (CN ranging from 3 to 4.9); AMPLIFICATION: LRR ≥ 1.3 (CN≥4.9). (B) Bar plot showing the three CN categories we identified by using the thresholds described above


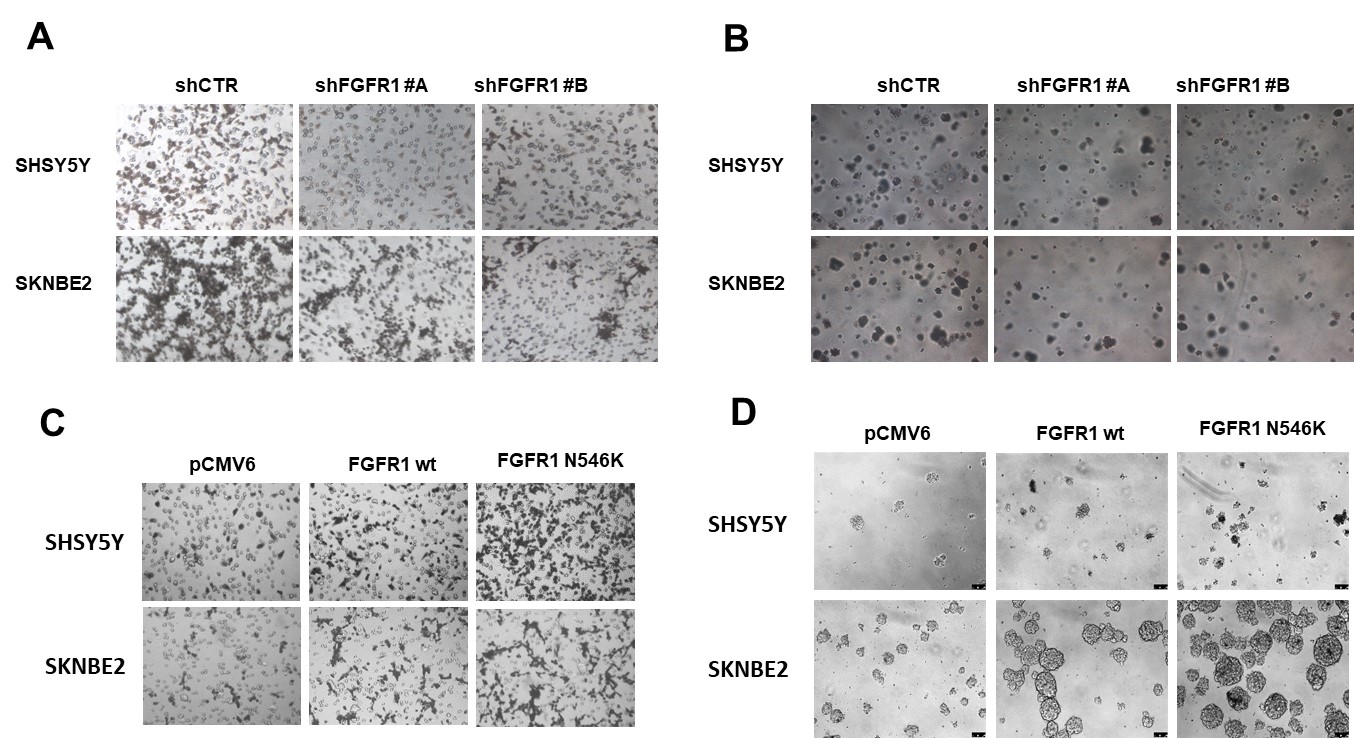


**Figure S2** Representative images of (A) invasion and (B) soft-agar assays in silenced *FGFR1* SHSY5Y and SKNBE2 cells. (C) Invasion and (D) neurosphere assays in *FGFR1* overexpressing cells are shown


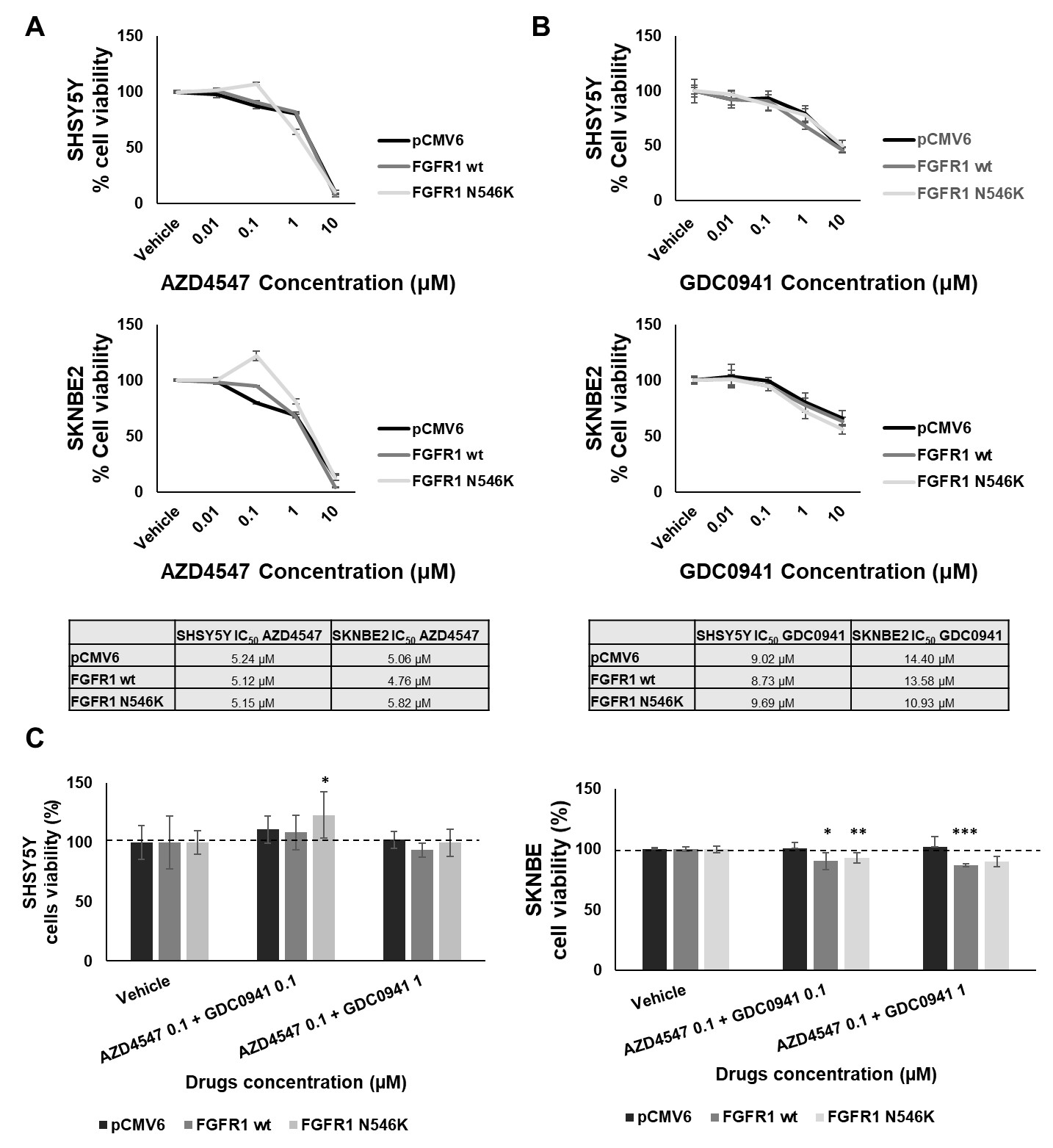


**Figure S3** SH5YSY and SKNBE2 cells were transiently transfected with pCMV6- empty vector, pCMV6- -FGFR1^wt^, pCMV6- - FGFR1^N546K^.In these cell lines, MTT assay to determine the IC50 value of (A) AZD4547 and (B) GDC0941 to analyze their effect on cell viability (%). The IC50 value (that is, the concentration of drug which exhibited 50% cell) are reported in table under the corresponding graph. (C) Bar plot represented the cell viability (%) of described above cell lines treated with different concentrations of AZD4547 and GDC0941 combination. Vehicle=DMSO. * = *p*-value ≤ 0.05, ** = *p-*value ≤ 0.01, *** = *p-*value ≤ 0.001


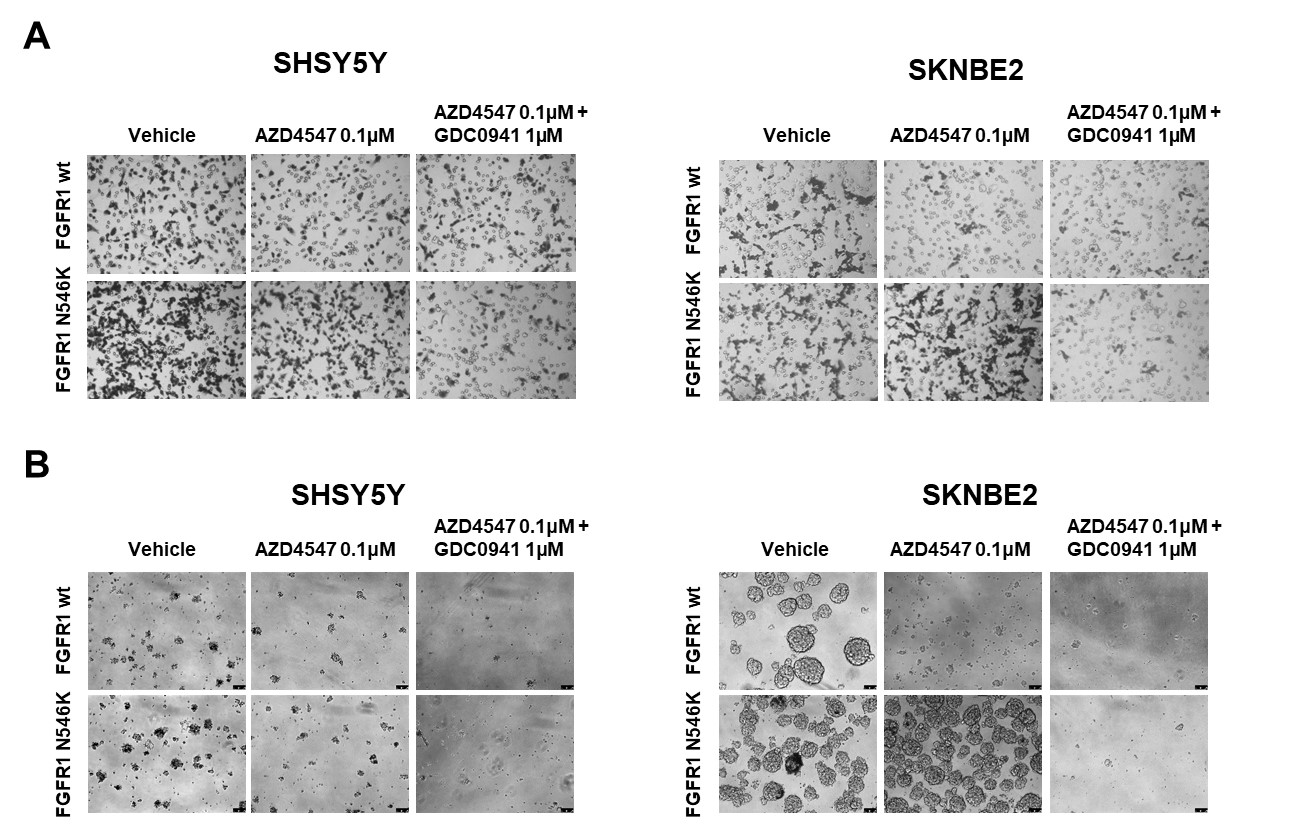


**Figure S4** Representative images of (A) the invasion assay and (B) neurospheres assay in SHSY5Y and SKNBE2 overexpressing FGFR1^wt^ or FGFR1^N546K^ cells after treatment with AZD4547 0.1µM alone and in combination with GDC0941 1µM. Vehicle=DMSO
